# Cytochrome P450-mediated metabolism of *N*-(2-methoxyphenyl)-hydroxylamine, a human metabolite of the environmental pollutants and carcinogens *o*-anisidine and *o*-nitroanisole

**DOI:** 10.2478/v10102-010-0045-8

**Published:** 2010-11

**Authors:** Karel Naiman, Helena Dračínská, Martin Dračínský, Markéta Martínková, Václav Martínek, Petr Hodek, Martin Štícha, Eva Frei, Marie Stiborová

**Affiliations:** 1 Department of Biochemistry, Faculty of Science, Charles University, Prague, Czech Republic; 2 Institut of Organic Chemistry and Biochemistry, v.v.i., Academy of Sciences, Prague, Czech Republic; 3 Department of Organic Chemistry, Faculty of Science, Charles University, Prague, Czech Republic; 4 Division of Molecular Toxicology, German Cancer Research Center, Heidelberg, Germany

**Keywords:** *o*-anisidine, *N*-(2-methoxyphenyl)hydroxylamine, metabolism, oxidation, cytochrome P450

## Abstract

*N*-(2-methoxyphenyl)hydroxylamine is a human metabolite of the industrial and environmental pollutants and bladder carcinogens 2-methoxyaniline (*o*-anisidine) and 2-methoxynitrobenzene (*o*-nitroanisole). Here, we investigated the ability of hepatic microsomes from rat and rabbit to metabolize this reactive compound. We found that *N*-(2-methoxyphenyl)hydroxylamine is metabolized by microsomes of both species mainly to *o*-aminophenol and a parent carcinogen, *o*-anisidine, whereas 2-methoxynitrosobenzene (*o*-nitrosoanisole) is formed as a minor metabolite. Another *N*-(2-methoxyphenyl)hydroxylamine metabolite, the exact structure of which has not been identified as yet, was generated by hepatic microsomes of rabbits, but its formation by those of rats was negligible. To evaluate the role of rat hepatic microsomal cytochromes P450 (CYP) in *N*-(2-methoxyphenyl)hydroxylamine metabolism, we investigated the modulation of its metabolism by specific inducers of these enzymes. The results of this study show that rat hepatic CYPs of a 1A subfamily and, to a lesser extent those of a 2B subfamily, catalyze *N*-(2-methoxyphenyl)hydroxylamine conversion to form both its reductive metabolite, *o*-anisidine, and *o*-aminophenol. CYP2E1 is the most efficient enzyme catalyzing conversion of *N*-(2-methoxyphenyl)hydroxylamine to o-aminophenol.

## Introduction

2-Methoxyaniline (*o*-anisidine) is a potent carcinogen, causing tumors of the urinary bladder in both genders of F344 rats and B6C3F1 mice (NTP, 1978; IARC, 1982). The International Agency for Research on Cancer (IARC) has classified *o*-anisidine as a group 2B carcinogen (IARC, 1982), which is possibly carcinogenic to humans. Besides its carcinogenicity it exhibits other toxic effects, including hematological changes, anemia and nephrotoxicity (NTP, 1978; IARC, 1982). *o*-Anisidine is used as an intermediate in the manufacturing of a number of azo and naphthol pigments and dyes, which are used for printing (90%) and for paper (3%) and textile (7%) dyeing (NTP, 1978; Garner *et al*., 1984). Such a wide use of this aromatic amine could result in occupational exposure. Furthermore, it may be released from textiles and leather goods colored with these azo dyes and a large part of the population may be exposed. This carcinogen is also a constituent of cigarette smoke (IARC, 1982; Stabbert *et al*., 2003). This strongly suggests that *o*-anisidine ranks not only among occupational pollutants produced in the manufacturing of chemicals, but also among environmental pollutants; it can be assumed that human exposure is widespread. Indeed, *o*-anisidine was found in human urine samples in the general population, in concentrations of 0.22 µg/l (median) (Weiss and Angerer, 2003). In addition, hemoglobin adducts of *o*-anisidine were detected in blood samples of persons living in urban or rural areas of Germany (Falter *et al*., 1994; Branner *et al*., 1998; Richter *et al*., 2001). The adducts as well as *o*-anisidine in urine might originate not only from the sources mentioned above, but also from a possible *o*-anisidine precursor, 2-methoxynitrobenzene (*o*-nitroanisole). This chemical was released into the environment in the course of an accident in a German chemical plant, causing subsequently local and regional contamination (Falter *et al*., 1994; Hauthal, 1993; Traupe *et al*., 1997). *o*-Nitroanisole exhibits strong carcinogenic activity, causing neoplastic transformation in the urinary bladder, and to a lesser extent, in the spleen, liver and kidneys in rodents (NTP 1993). It is also a toxic compound, causing anemia. The anemia is characterized by increased levels of methemoglobin and accelerated destruction of erythrocytes (NTP 1993).

Recently, we have found that *o*-anisidine is oxidatively activated by peroxidase and cytochrome P450 (CYP) to species binding to DNA *in vitro* (Stiborová *et al*., 2001; 2002; 2005; Rýdlová *et al*., 2005; Naiman *et al*., 2008). We also demonstrated that *o*-anisidine forms DNA adducts *in vivo*. The same adducts as found in DNA incubated with *o*-anisidine and human microsomes *in vitro* were detected in urinary bladder, the target organ, and to a lesser extent, in liver, kidney and spleen of rats treated with *o*-anisidine (Stiborová *et al*., 2005). The *o*-anisidine-derived DNA adducts were identified as deoxyguanosine adducts formed from a metabolite of *o*-anisidine, *N*-(2-methoxyphenyl)hydroxylamine, which is generated by oxidation of *o*-anisidine with human, rabbit and rat hepatic microsomes (Stiborová *et al*., 2005; Rýdlová *et al*., 2005; Naiman *et al*., 2008). The same deoxyguanosine adducts were also detected in DNA of the urinary bladder, kidney, liver and spleen of rats treated with *o*-nitroanisole (Stiborová *et al*., 2004), an oxidized counterpart of *o*-anisidine, and in DNA incubated with *o*-nitroanisole *in vitro* with human and rat hepatic cytosolic enzymes and xanthine oxidase (Stiborová *et al*., 1998; 2004). These enzymatic systems were found to produce *N*-(2-methoxyphenyl)hydroxylamine after *o*-nitroanisole reduction (Mikšanová *et al*., 2004). The data indicate that formation of *N*-(2-methoxyphenyl)hydroxylamine, the reactive metabolite of both carcinogens, is critical for generation of DNA lesions in target organs. Therefore, it is clear that *N*-(2-methoxyphenyl)hydroxylamine formation and its further conversion, as well as the enzymes participating in such processes, play a key role in carcinogenic effects of both carcinogens.

**Figure 1:**
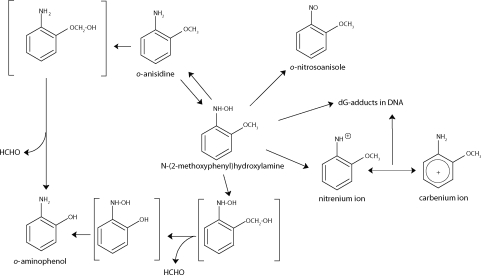
Pathways of *o*-anisidine metabolism by the cytochrome P450 system showing the characterized metabolites and those proposed to form DNA adducts. The compounds shown in brackets were not detected under the experimental conditions.

Recently, we have found that *o*-anisidine is oxidized by human, rat and rabbit hepatic microsomes not only to *N*-(2-methoxyphenyl)hydroxylamine, but that this compound is a subject of complex redox cycling reactions, forming also *o*-aminophenol, *o*-nitrosoanisole and one additional metabolite, the exact structure of which has not been identified as yet (Stiborová *et al*., 2005; Naiman *et al*., 2008). *N*-(2-methoxyphenyl)hydroxylamine might also be a subject of complex reactions, and its fate is dependent on the environment, in which it occurs. It can be further metabolized to *o*-aminophenol, *o*-nitrosoanisole and parental *o*-anisidine (Naiman *et al*., 2008), or when nucleophiles such as DNA or proteins are present in the cell, form the adducts (Stiborová *et al*., 2005) (Figure 1).

The results of our former studies (Stiborová *et al*., 2005; Naiman *et al*., 2008) show a similarity among hepatic microsomal CYP systems metabolizing *o*-anisidine in humans and two animal models, rats and rabbits, which are the species, in which this agent is carcinogenic or toxic (NTP, 1978; IARC, 1982). These findings indicate that both experimental animal species might serve as suitable models to mimic the fate of this carcinogen in human. However, whereas formation of DNA adducts by *N*-(2-methoxyphenyl)hydroxylamine, generated in reactions catalyzed with human and rat hepatic microsomes, has been clearly proven (Stiborová *et al*., 2005), conversion of this metabolite to further products has been investigated using only rabbit hepatic microsomes (Naiman *et al*., 2008). Therefore, the aim of the present study was to evaluate the efficiency of *N*-(2-methoxyphenyl)hydroxylamine metabolism by hepatic microsomes from rats.

## Materials and methods

### Chemicals

Chemicals were obtained from the following sources: β-naphthoflavone (β-NF), NADP^+^, NADPH, glucose 6-phosphate and bicinchoninic acid (2,2′-biquinoline-4,4′-dicarboxylic acid) from Sigma Chemical Co. (St. Louis, MO, USA); *o*-anisidine, *o*-aminophenol (>99% based on HPLC) from Fluka Chemie AG (Buchs, Switzerland) and glucose 6-phosphate dehydrogenase from Serva (Heidelberg, Germany). All these and other chemicals were of analytical purity or better. 2-Methoxynitrosobenzene was synthesized in analogy to the synthesis described earlier (Seidenfaden, 1971) by oxidation of *N*-(2-methoxyphenyl)hydroxylamine with potassium dichromate in water and identified by ^1^H NMR recorded on a 400 MHz instrument in CDCl_3_ (referenced to TMS): 7.68 (1H, m), 7.36 (1H, m), 6.86 (1H, m), 6.30 (1H, m) and 4.28 (3H, s). *N*-(2-methoxyphenyl)hydroxylamine was synthesized by the procedure similar to that described earlier (Balaban *et al*., 1998). *N*-(2-Methoxyphenyl)hydroxylamine authenticity was confirmed by electrospray mass and CID spectra and high field proton NMR spectroscopy. The positive-ion electrospray mass-spectrum exhibited the protonated molecule at *m*/*z* 140.1, while the CID of its ion fragments at *m*/*z* 125.2, 108.1 and 109.1. The ^1^H-NMR spectra were recorded at 400 MHz in dimethyl sulfoxide-*d*_*6*_. The central line of dimethyl sulfoxide at 2.500 ppm was used as reference line. The spectra showed the presence of the following protons: 8.28 (1H, d, J = 2.3 Hz, exchanged with CD_3_OD), 7.64 (1H, d, J = 1.5 Hz, exchanged with CD_3_OD), 7.01 (1H, m, ∑ J = 9.6 Hz), 6.84 (2H, m, ∑ J = 15.0 Hz), 6.75 (1H, m, ∑ J = 16.9 Hz), 3.75 (3H, s). *o*-Aminophenol authenticity was confirmed by ESI mass spectra and ^1^H and ^13^C NMR spectroscopy. The positive-ion mass-spectrum exhibited the protonated molecule at *m/z* 110.4, while the negative-ion mass-spectrum the molecule at *m/z* 108.4. The NMR spectra were recorded on a Bruker Avance II-500 instrument (499.8 MHz for ^1^H and 125.7 MHz for ^13^C) in dimethyl sulfoxide-*d*_*6*_ and referenced to the solvent signal (δ 2.50 and 39.70, respectively). ^1^H NMR spectrum showed the presence of the following signals: 8.96 (1H, bs), 6.65 (1H, m), 6.59 (1H, m), 6.54 (1H, m), 6.40 (1H, m) and 4.46 (2H, bs). ^13^C NMR spectrum showed the presence of six signals of aromatic carbons: 144.26 (s), 136.78 (s), 119.80 (d), 116.75 (d), 114.73 (d) and 114.64 (d).

### Animal experiments, preparation of microsomes and assays

The study was conducted in accordance with the Regulations for the Care and Use of Laboratory Animals (311/1997, Ministry of Agriculture, Czech Republic), which complies with Declaration of Helsinki. Microsomes from livers of ten untreated rats and three rabbits were prepared by the procedure described previously (Stiborová *et al*., 1988). Microsomes from the livers of ten male Wistar rats pretreated with β-NF (Stiborová *et al*., 1988) were isolated as described (Stiborová *et al*., 1988; 1995), those pretreated with phenobarbital (PB) as reported by Hodek *et al*. (1988), and those pretreated with ethanol were isolated using a procedure described by Yang *et al*. (1985). Protein concentrations in the microsomal fractions were assessed using the bicinchoninic acid protein assay with bovine serum albumin as a standard (Wiechelman *et al*., 1988). The concentration of CYP was estimated according to Omura and Sato (1964), by measuring the absorption of the complex of reduced CYP with carbon monoxide. Rat and rabbit liver microsomes contained 0.6 and 1.8 nmol CYP/mg protein, respectively. Hepatic microsomes of rats induced with β-NF, PB, and ethanol contained 1.3, 2.7 and 1.8 nmol CYP/mg protein, respectively. The activity of NADPH:CYP reductase in rat hepatic microsomes was measured according to Sottocasa *et al*. (1967) using cytochrome c as substrate (i.e., as NADPH:cytochrome c reductase). NADPH:CYP reductase activities in hepatic microsomes of control (uninduced) rats and those induced with β-NF, PB and ethanol were 0.210, 0.199, 0.325 and 0.201 µmol/min/mg protein, respectively.

### Incubations

Incubation mixtures used for study of the *o*-anisidine metabolism contained the following concentrations in a final volume of 100 µl: 100 mM sodium phosphate buffer (pH 7.4), 1 mM NADP^+^, 10 mM D-glucose 6-phosphate, 1 U/ml D-glucose 6-phosphate dehydrogenase (NADPH-generation system), a rat or rabbit hepatic microsomal fraction containing 0.04–1.0 nmol CYP, and 0.1–2.0 mM *o*-anisidine dissolved in 1.0 µl methanol. The reaction was initiated by adding the substrate. To study metabolism of *N*-(2-methoxyphenyl)hydroxylamine, incubation mixtures contained the following concentrations in a final volume of 100 µl: 100 mM sodium phosphate buffer (pH 7.4), 1 mM NADP^+^, 10 mM D-glucose 6-phosphate, 1 U/ml D-glucose 6-phosphate dehydrogenase (NADPH-generation system), a rat and rabbit hepatic microsomal fraction containing 0.04–1.0 nmol CYP, and 0.1–1.0 mM *N*-(2-methoxyphenyl)hydroxylamine dissolved in 1.0 µl distilled water. The reaction was initiated by adding the substrate. After incubation in open glass tubes (37^o^C, 30 min), the reactions were terminated by adding 100 µl of methanol and centrifuged at 5,000 g for 5 min. Metabolism of *o*-anisidine and *N*-(2-methoxyphenyl)hydroxylamine with rat and rabbit CYP enzymatic systems was linear until 40 min. The supernatants were collected and 20 µl aliquots applied onto a high-performance liquid chromatography (HPLC) column, where metabolites of *o*-anisidine were separated. The HPLC was performed on a C-18 reversed-phase column (250 × 4.6 mm, 5 µm, Nucleosil 100-5, Macherey-Nagel, Duren, Germany). Metabolites were eluted with 18% methanol, 82% 7.18 µM aqueous ammonia, pH 8.0, (v/v) at a flow rate of 0.6 ml/min and monitored at 254 nm. *o*-Anisidine and *N*-(2-methoxyphenyl)hydroxylamine metabolites were analyzed by mass spectrometry and by comparing their chromatographic properties on HPLC with those of synthetic standards. 2-Methoxynitrosobenzene, *o*-aminophenol, *N*-(2-methoxyphenyl)hydroxylamine and 2-methoxynitrobenzene standards, were eluted at retention times (r.t.) of 8.8, 11.3, 19.7 and 57.5 min, respectively (Stiborová *et al*., 2005; Naiman *et al*., 2008).

## Results

### Metabolism of *N*-(2-methoxyphenyl)hydroxylamine by rat and rabbit hepatic microsomes

**Figure 2:**
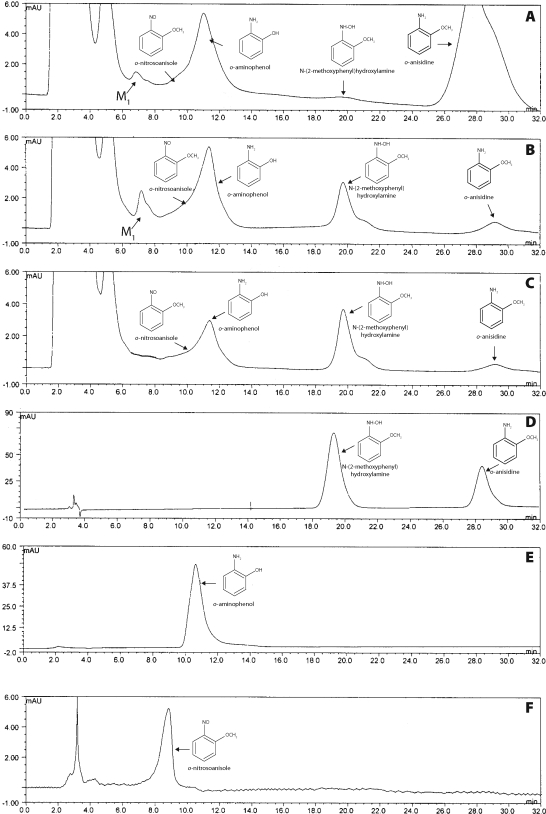
HPLC elution profiles of metabolites of 1 mM *o*-anisidine incubated with rabbit microsomes (**A**), of 1.0 mM *N*-(2-methoxyphenyl)hydroxylamine incubated with rabbit (**B**) and rat (**C**) hepatic microsomes. (**D**) synthetic *N*-(2-methoxyphenyl)hydroxylamine and *o*-anisidine. (**E**) *o*-aminophenol. (**F**) *o*-nitrosoanisole. For incubation conditions see *Materials and methods*. Peaks eluting between 2.0 and 5.5 min, solvent front, NADPH and protein components of microsomes and NADPH-generation system.

When a parental compound from which *N*-(2-methoxyphenyl)hydroxylamine is generated, *o*-anisidine, was incubated with rat and rabbit hepatic microsomes in the presence of NADPH, two metabolites, *o*-aminophenol (r.t. of 11.3 min) and *N*-(2-methoxyphenyl)hydroxylamine (r.t. of 19.7 min), as well as an additional product peak of metabolite 1 (M1, r.t. of 7.0 min), which structure has not been identified as yet, were separated by HPLC (see peaks in Figure 2A for the profile obtained with rabbit microsomes) (Naiman *et al*., 2008).

The results of experiments with *N*-(2-methoxyphenyl)hydroxylamine and microsomes demonstrated that metabolites M1 and *o*-aminophenol are also formed from this *o*-anisidine metabolite by rabbit hepatic microsomes (Figure 2B), while formation of the metabolite M1 from this compound by hepatic microsomes of rats was negligible (Figure 2C). When *N*-(2-methoxyphenyl)hydroxylamine was incubated without hepatic microsomal enzymes or without NADPH, metabolite M1 and *o*-aminophenol peaks were also detectable by HPLC, but only under acidic conditions (pH 4.5 for 60 min). At pH 7.4, used for microsomal incubations, their spontaneous formation was negligible. This finding indicates that conversion of *N*-(2-methoxyphenyl)hydroxylamine in microsomes is mediated by enzymatic reactions. During metabolism of *N*-(2-methoxyphenyl)hydroxylamine by hepatic microsomes of both species, a shoulder at 8.8 min was also detectable (Figure 2B,C), suggesting formation of *o*-nitrosoanisole (2-methoxynitrosobenzene) (r.t. of 8.8 min, Figure 2F). Moreover, during the incubations of *N*-(2-methoxyphenyl)hydroxylamine with rat and rabbit microsomes and NADPH, an additional product peak was detected by HPLC, being identified to be the parental compound, *o*-anisidine (r.t. of 28.6 min) (Figure 2B,C).

### Involvement of CYP enzymes in *N*-(2-methoxyphenyl)hydroxylamine metabolism in rat hepatic microsomes

In order to evaluate the participation of individual rat hepatic microsomal CYPs in *N*-(2-methoxyphenyl)hydroxylamine metabolism, the induction of individual CYP enzymes was performed with this animal model. Microsomes isolated from livers of uninduced rats and rats pre-treated with β-NF (enriched with CYP1A1/2), PB (enriched with CYP2B1/2) and ethanol (enriched with CYP2E1) were used (Table 1).

Except of the enzymes present in microsomes of livers of rats pre-treated with ethanol, the enzymes of all other microsomes tested in this study formed from *N*-(2-methoxyphenyl)hydroxylamine predominantly its reductive metabolite, *o*-anisidine. More than 2-fold higher levels of *o*-anisidine than *o*-aminophenol were determined when *N*-(2-methoxyphenyl)hydroxylamine was incubated with these microsomes and NADPH (Table 1). Levels of the *N*-(2-methoxyphenyl)hydroxylamine reductive metabolite, *o*-anisidine, did not correspond to activities of NADPH:CYP reductase in individual microsomes. Namely, activities of this enzyme in hepatic microsomes of control (uninduced) rats and those induced with β-NF, PB and ethanol used in the experiments are similar, being 0.210, 0.199, 0.325 and 0.201 µmol/min/mg protein, respectively. While incubations of *N*-(2-methoxyphenyl)hydroxylamine with hepatic microsomes of rats pre-treated with β-NF led to a 2.4- and 1.9-fold increase (*P*<0.05) in *o*-aminophenol and *o*-anisidine formation, respectively (Table 1), another inducer of CYP enzymes, PB, had much less effect. Even though a 1.4- and 1.2-fold increase in *o*-aminophenol and *o*-anisidine formation, respectively, was mediated by treating rats with this CYP inducer (Table 1), this increase was statistically insignificant. Ethanol, an inducer of CYP2E1, stimulated production of *o*-aminophenol from *N*-(2-methoxyphenyl)hydroxylamine, by 3-fold (*P*<0.05), whereas decreased levels of the *N*-(2-methoxyphenyl)hydroxylamine reduction metabolite, *o*-anisidine. These results indicate that rat hepatic CYP2E1, CYPs of a 1A subfamily and, to a lesser extent, those of a 2B subfamily, are capable of metabolizing *N*-(2-methoxyphenyl)hydroxylamine in rat livers.

**Table 1:** Metabolism of *N*-(2-methoxyphenyl)hydroxylamine in rat hepatic microsomes induced with different agents

Hepatic microsomes	*N*-(2-methoxyphenyl)hydroxylamine metabolites^a^	
from rats pretreated with^b^	*o*-aminophenol	*o*-anisidine
None-control microsomes	2.0 ± 1.0	5.8 ± 0.7
β-naphthoflavone (CYP1A1/2)	4.8 ± 1.7	11.1 ± 3.3
Phenobarbital (CYP2B1/2)	2.8 ± 2.5	7.0 ± 2.0
Ethanol (CYP2E1)	6.1 ± 3.1	3.5 ± 0.7

a The numbers are the peak area/min/nmol CYP for each metabolite; averages ± S.E.M of three determinations in separate experiments.

b Isoforms of CYP induced are shown in brackets.

It should be noted that the results of experiments with inducers should be carefully interpreted, because these inducers are not absolutely specific for individual CYPs. Therefore, to confirm the role of these CYPs in *N*-(2-methoxyphenyl)hydroxylamine metabolism, additional experimental approaches such as selective inhibition of CYPs and utilization of the purified CYP reconstituted with NADPH: CYP reductase are planned to be employed in further studies.

## Discussion

The results of this study show that rat and rabbit hepatic microsomes can metabolize *N*-(2-methoxyphenyl)hydroxylamine, a reactive metabolite of carcinogenic *o*-anisidine and *o*-nitroanisole. This compound is responsible for genotoxic effects of both carcinogens, because it is easily decomposed to the nitrenium/carbenium ion forming DNA adducts (Figure 1) (Stiborová *et al*., 2004; 2005; Naiman *et al*., 2008). The results demonstrate that *N*-(2-methoxyphenyl)hydroxylamine is also further metabolized to *o*-aminophenol, *o*-nitrosoanisole and the parent compound, *o*-anisidine. The formed *o*-anisidine may be *O*-demethylated again to *o*-aminophenol (Figure 1). The question whether *o*-aminophenol is also formed from *N*-(2-methoxyphenyl)hydroxylamine by its *O*-demethylation to *N*-(2-hydroxyphenyl)hydroxylamine, which is subsequently reduced to *o*-aminophenol (Figure 1), remains to be answered. No metabolites formed by this reaction were observed.

Recently, redox cycling reactions similar to those we found with *N*-(2-methoxyphenyl)hydroxylamine were observed by Kim *et al*. (2004), who studied metabolism of several aromatic and heterocyclic amines by a CYP1A2/NADPH:CYP reductase enzymatic system. They reported that the CYP system catalyzes oxidation of the *N*-hydroxylated intermediate formed from the carcinogenic heterocyclic amine 2-amino-3-methylimidazo[4,5-*f*]quinoline (IQ), to a nitrosoderivative. They demonstrated that NADPH:CYP reductase can catalyze the reduction of the IQ oxidation products, *N*-nitroso-IQ and *N*-hydroxyl-IQ, to *N*-hydroxyl-IQ and the parent amine, IQ (Kim *et al*., 2004). *N*-hydroxylation products of two other aromatic amines investigated by Kim *et al*. (2004), 2-aminofluorene and 4-aminobiphenyl, are, however, reduced non-enzymatically, by NADPH. We have not determined whether reduction of *N*-(2-methoxyphenyl)hydroxylamine to *o*-anisidine requires catalysis by NADPH:CYP reductase or occurs non-enzymatically, or is mediated by other enzymes. However, preliminary experiments performed in our laboratory suggest that although NADPH:CYP reductase might partially participate in *N*-(2-methoxyphenyl)hydroxylamine reduction to *o*-anisidine, CYP enzymes present in hepatic microsomes are more effective in this process. (Naiman *et al*., unpublished results). Indeed, the results of the present study demonstrate that rat hepatic microsomes reduce *N*-(2-methoxyphenyl)hydroxylamine to *o*-anisidine independently on activities of NADPH:CYP reductase. The present study shed also some light on the role of specific microsomal CYP enzymes in metabolism of *N*-(2-methoxyphenyl)hydroxylamine. The CYP1A enzymes seem to be most efficient in *N*-(2-methoxyphenyl)hydroxylamine reduction. Nevertheless, the question whether CYP2E1 is also effective in reducing *N*-(2-methoxyphenyl)hydroxylamine, remains to be answered. Among hepatic microsomes tested in this study, those of rats treated with ethanol (enriched with CYP2E1) produced the lowest levels of *o*-anisidine from *N*-(2-methoxyphenyl)hydroxylamine. In contrast, *o*-aminophenol was the major metabolite in microsomes enriched with CYP2E1, generated at the highest levels among the microsomes employed in the experiments. These findings suggest that CYP2E1 is not effective in *N*-(2-methoxyphenyl)hydroxylamine reduction. Nevertheless, because CYP2E1 is the most effective CYP enzyme oxidizing *o*-anisidine to *o*-aminophenol (Stiborová *et al*., 2005; Naiman *et al*., 2008), one can speculate that this enzyme efficiently utilizes *o*-anisidine, which could be generated in this microsomal system, oxidizing it to *o*-aminophenol. Therefore, the participation of CYP2E1 in *N*-(2-methoxyphenyl)hydroxylamine reduction to *o*-anisidine cannot be excluded and awaits further investigation. The study utilizing purified CYP2E1 reconstituted with NADPH:CYP reductase is planned to be performed to explain this question.

While the formation of *N*-(2-methoxyphenyl)hydroxylamine was clearly identified to be the activation pathway of *o*-anisidine and *o*-nitroanisole metabolism (Stiborová *et al*., 2004; 2005), biological significance of formation of *o*-aminophenol for detoxication/activation metabolism awaits further investigation. *o*-Aminophenol might be considered to be mutagenic, because it induces sister chromatid exchanges in a dose-dependent manner in cultured human lymphocytes *in vitro* and in Chinese hamster bone marrow cells *in vivo* (Kirchner and Bayer, 1992). In addition, Brennan and Schiestl (1997) reported that *o*-aminophenol is positive in the deletion recombination assay in *Sacchromyces cerevisiae*. Even though *o*-aminophenol has not been found to form covalent DNA adducts, it was demonstrated in *in-vitro* experiments to cause DNA damage, forming 8-oxy-7,8-dihydro-2′-deoxyguanosine in the presence of metal ions such as Cu(II) (Okhuma and Kawanishi, 2001). Hence, due to such processes, *o*-aminophenol may contribute to initiation of the *o*-anisidine- and/or *o*-nitroanisole-mediated carcinogenesis in the urinary bladder, and in a tumor development induced by other bladder carcinogenic aromatic amines, which produce this compound as one of the metabolites (Brennan and Schiestl, 1997). Furthermore, *O*-demethylation reactions produce formaldehyde (Figure 1), which is known to modify DNA, generating several products including hydroxymethyl adducts and cross-links (Beland *et al*., 1984; Huang and Hopkins, 1993; Cheng *et al*., 2003). Formaldehyde is mutagenic in a variety of different test systems and carcinogenic in laboratory animals (IARC, 2006) and has been described as “carcinogenic to human” by the IARC and “reasonably anticipated to be a human carcinogen” by the U.S. Department of Health and Human Services (2004). Therefore, on the one hand, it is plausible that formaldehyde-DNA adducts could also play a role in carcinogenesis by *o*-anisidine. On the other hand, however, formaldehyde produced in the cell is also detoxified by conjugation to glutathione and oxidized (Dhareshwar and Stella, 2008); therefore, it is not likely a strong contributor to carcinogenicity caused by *o*-anisidine.

It should be noted that tumor development in a specific organ is influenced by promotional pressures on initiated cells in target organs and not only by the levels of DNA adducts formed by the compounds like *o*-anisidine and *o*-nitroanisole. It is known that radicals formed from several carcinogens producing oxidative DNA damage, such as 8-hydroxy-2′-deoxyguanosine, are important not only in initiation, but also in promotion phases of carcinogenesis (Imaoka *et al*., 2004). Therefore, their formation from *o*-aminophenol may be one of the factors contributing to tumor promotion in *o*-anisidine- and/or *o*-nitroanisole-mediated carcinogenesis. In addition, *o*-anisidine is oxidized by several peroxidases, which are expressed in target organs (e.g. COX), to form radicals besides DNA adducts (Brennan, and Schiestl, 1999; Sasaki *et al*., 1998). Hence, the production of such free radicals in or near the target cells may be another factor important in the promotional process in *o*-anisidine-mediated tumor development. However, the exact functions of such and/or other promotional pressures caused both by *o*-anisidine and *o*-aminophenol in an *o*-anisidine-mediated tumorigenesis remain to be resolved.
